# LG biplot: a graphical method for mega-environment investigation using existing crop variety trial data

**DOI:** 10.1038/s41598-019-43683-9

**Published:** 2019-05-09

**Authors:** Weikai Yan

**Affiliations:** Ottawa Research and Development Center, Agriculture and Agri-Food Canada, Building 20, 960 carling Ave., Ottawa, Ontario, KA1 0C6 Canada

**Keywords:** Plant breeding, Data mining

## Abstract

Due to the presence of genotype by environment interaction (GE), no crop cultivar performed the best in all regions. Therefore, the growing regions of a crop must be divided into sub-regions or mega-environments, and specifically adapted cultivars must be bred and deployed in each mega-environment. Meaningful mega-environment delineation must be based on repeatable GE patterns, which can be extracted from multi-year, multi-location crop variety trials. In regional crop variety trials, usually the same set of genotypes are tested across locations within a year, but different sets of genotypes are tested in different years, leading to highly unbalanced multi-year data. Such data are abundant for all crops and regions; but there has been no way to fully utilize them for mega-environment delineation. This paper presents a new method that allows utilization of existing variety trial data to identify repeatable GE patterns, to delineate mega-environments, and to understand the scope of unrepeatable GE at a location and within a mega-environment.

## Introduction

Plant breeding has played a key role in increasing agricultural productivity and feeding the world. One key aspect of plant breeding is to select and deploy specifically adapted crop cultivars to a target region. Due to the presence of genotype by environment interaction (GE), no crop cultivar performs the best in all environments. To deal with GE, the growing regions of a crop must be divided into subregions, i.e., mega-environments^1^. Although there are many ways to delineate a target region or regions into mega-environments, meaningful mega-environment delineation must be based on repeatable GE patterns, which can be extracted only from multi-year, multi-location crop variety trials^2,3^. Unfortunately, although multi-location variety trials are conducted every year in every region for all major crops, data from such trials have rarely been utilized to extract repeatable GE patterns, because different sets of genotypes are tested in different years and multi-year data are highly unbalanced. Some attempts have been made to utilize such data; the common strategy has been to analyze multi-location variety trials yearly and to summarize the patterns across years^4^. The main technique has been GGE (genotypic main effect plus GE) biplot analysis^5–8^, which was developed based on the biplot theory of Gabriel^9^. Summarizing GE patterns across years proves to be very tedious and difficult. Recently, a GGE-GGL biplot was developed, which allows multi-year, multi-location crop variety trial data to be displayed in a single GGE biplot and thereby allows clear separation of repeatable GE from unrepeatable GE^2^. The GGE-GGL biplot method requires a sizable number of common genotypes to be present across years so that missing values can be imputed with confidence; this requirement is not always met in routinely conducted crop variety trials, however. The objective of this paper is to present a location-grouping (LG) biplot method for mega-environment delineation that does not require common genotypes across years so that data from routinely conducted variety trials can be utilized to understand the mega-environments and test locations for any crop and region.

## Results

### The GGE biplots to show yearly GE patterns

**Figure 1:**
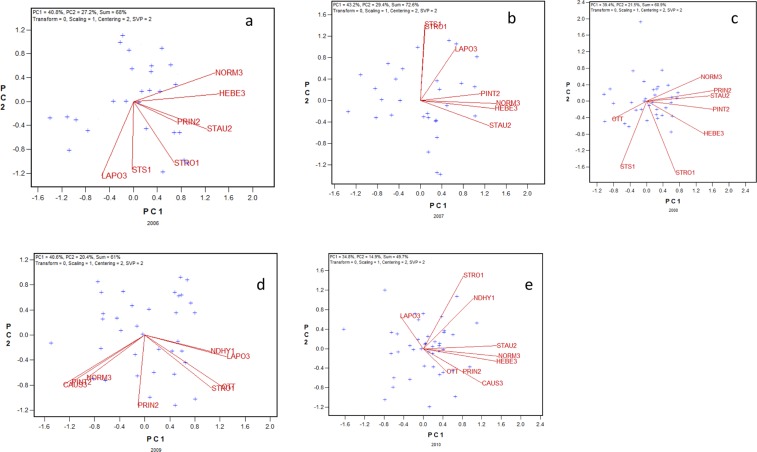
The GGE (genotypic main effect plus genotype-by-environment interaction) biplot for yield for each of the five years from 2006 to 2010. The biplots were based on location standardized data (Scaling = 1, Centering = 2) and the singular values were partitioned entirely to the location vectors to focus on the relations among test locations (SVP = 2). The genotypes are shown as “+” for clarity. See Table 1 for the full location names.

The yearly GGE biplots summarizing the grain yield data from the 2006 to 2010 Quebec Provincial Oat Registration and Recommendation trials are presented in Fig. 1. Although a GGE biplot can be visualized in many ways to address different questions^10^, the focus here is to visualize the similarity/dissimilarity among test locations in ranking genotypes. The genotypes tested each year were considered as random samples of the genotype population so their names were not spelled out for clarity. The GGE biplot were based on location-standardized data (“Scaling = 1”, “Centering = 2”) and the singular values were partitioned entirely to the location vectors (“SVP = 2”). These settings (shown on the upper left corner of the biplots) allow the following interpretation: the cosine of the angle between two locations in the biplot approximates the Pearson correlation between them across tested genotypes (Table 1). The closeness of the approximation is related to the goodness of fit of the biplot, which is also shown on the upper left corner of each biplot (Fig. 1). An acute angle means positive correlation, an obtuse angle means negative correlation, and a right angle means lack of correlation between two locations across tested genotypes for the trait of interest (here grain yield). Both Fig. 1 and Table 1 can be used to understand the yearly relations among test locations. While the numerical values in Table 1 are more accurate, Fig. 1 allows a quick grasp of the main yearly patterns. From example, Fig. 1 shows that there were some negative correlations among locations (obtuse angles between locations) in each of the five years, indicating strong GE. The problem with both Table 1 and Fig. 1, however, is that it is difficult to extract the common patterns across years; that is, it is difficult to separate repeatable GE from unrepeatable GE, which is essential for meaningful mega-environment delineation.

**Table 1:** Pearson correlations between test locations across tested genotypes for grain yield in each of the years from the 2006 to 2010 Quebec provincial oat registration and recommendation trials.

Year	Locations^§^	CAUS3	HEBE3	LAPO3	NDHY1	NORM3	OTT	PINT2	PRIN2	STAU2	STRO1	STS1
2006	HEBE3	.	1	−0.330	.	0.870	.	.	0.359	0.670	0.310	−0.034
2006	LAPO3	.	−0.330	1	.	−0.499	.	.	−0.068	−0.008	0.363	0.435
2006	NORM3	.	0.870	−0.499	.	1	.	.	0.281	0.584	0.157	−0.200
2006	PRIN2	.	0.359	−0.068	.	0.281	.	.	1	0.236	0.205	0.223
2006	STAU2	.	0.670	−0.008	.	0.584	.	.	0.236	1	0.526	0.151
2006	STRO1	.	0.310	0.363	.	0.157	.	.	0.205	0.526	1	0.235
2006	STS1	.	−0.034	0.435	.	−0.2	.	.	0.223	0.151	0.235	1
2007	HEBE3	.	1	0.302	.	0.829	.	0.519	.	0.677	−0.084	−0.008
2007	LAPO3	.	0.302	1	.	0.431	.	0.132	.	0.054	0.339	0.452
2007	NORM3	.	0.829	0.431	.	1	.	0.479	.	0.701	−0.078	0.038
2007	PINT2	.	0.519	0.132	.	0.479	.	1	.	0.597	0.300	0.001
2007	STAU2	.	0.677	0.054	.	0.701	.	0.597	.	1	−0.156	−0.184
2007	STRO1	.	−0.084	0.339	.	−0.078	.	0.300	.	−0.156	1	0.705
2007	STS1	.	−0.008	0.452	.	0.038	.	0.001	.	−0.184	0.705	1
2008	HEBE3	.	1	.	.	0.419	−0.093	0.429	0.348	0.476	0.517	0.001
2008	NORM3	.	0.419	.	.	1	−0.002	0.425	0.432	0.466	−0.081	−0.336
2008	OTT	.	−0.093	.	.	−0.002	1	−0.298	−0.397	−0.284	0.027	0.183
2008	PINT2	.	0.429	.	.	0.425	−0.298	1	0.62	0.402	0.388	−0.200
2008	PRIN2	.	0.348	.	.	0.432	−0.397	0.62	1	0.543	0.186	−0.260
2008	STAU2	.	0.476	.	.	0.466	−0.284	0.402	0.543	1	0.206	−0.242
2008	STRO1	.	0.517	.	.	−0.081	0.027	0.388	0.186	0.206	1	0.476
2008	STS1	.	0.001	.	.	−0.336	0.183	−0.200	−0.260	−0.242	0.476	1
2009	CAUS3	1	.	−0.438	−0.387	0.486	−0.283	0.712	0.299	.	-0.251	.
2009	LAPO3	−0.438	.	1	0.321	−0.225	0.583	−0.411	−0.024	.	0.591	.
2009	NDHY1	−0.387	.	0.321	1	−0.267	0.573	−0.216	0.044	.	0.231	.
2009	NORM3	0.486	.	−−0.225	−0.267	1	−0.218	0.445	0.094	.	−0.070	.
2009	OTT	−0.283	.	0.583	0.573	−0.218	1	−0.184	0.153	.	0.589	.
2009	PINT2	0.712	.	−0.411	−0.216	0.445	−0.184	1	0.148	.	−0.250	.
2009	PRIN2	0.299	.	−0.024	0.044	0.094	0.153	0.148	1	.	0.190	.
2009	STRO1	−0.251	.	0.591	0.231	−0.07	0.589	−0.250	0.190	.	1	.
2010	CAUS3	1	0.521	−0.277	0.178	0.474	0.017	.	0.127	0.397	0.008	.
2010	HEBE3	0.521	1	−0.053	0.387	0.63	0.231	.	0.234	0.492	0.140	.
2010	LAPO3	−0.277	−0.053	1	−0.029	−0.021	−0.276	.	−0.025	−0.303	−0.008	.
2010	NDHY1	0.178	0.387	−0.029	1	0.316	0.050	.	0.080	0.296	0.498	.
2010	NORM3	0.474	0.63	−0.021	0.316	1	.	.	0.409	0.588	0.200	.
2010	OTT	0.017	0.231	−0.276	0.050	.	1	.	0.093	0.206	0.062	.
2010	PRIN2	0.127	0.234	−0.025	0.080	0.409	0.093	.	1	0.196	0.085	.
2010	STAU2	0.397	0.492	−0.303	0.296	0.588	0.206	.	0.196	1	0.383	.
2010	STRO1	0.008	0.140	−0.008	0.498	0.200	0.062	.	0.085	0.383	1	.

^§^The full names of the locations and their zones are: CAUS3: Causapscal (Zone-3), HEBE3: Hebertville (Zone-3), LAPO3: La Pocatière (Zone3), NORM3: Normandin (Zone-3), OTT: Ottawa (Ontario), PINT2: Pintendre (Zone-2), PRIN2: Princeville (Zone-2), STAU2: St-Augustin (Zone-2), STRO1: St- Rosalie (Zone-1), and STS1: St-Simon (Zone-1).

### The LG biplot to show repeatable and non-repeatable GE

**Figure 2:**
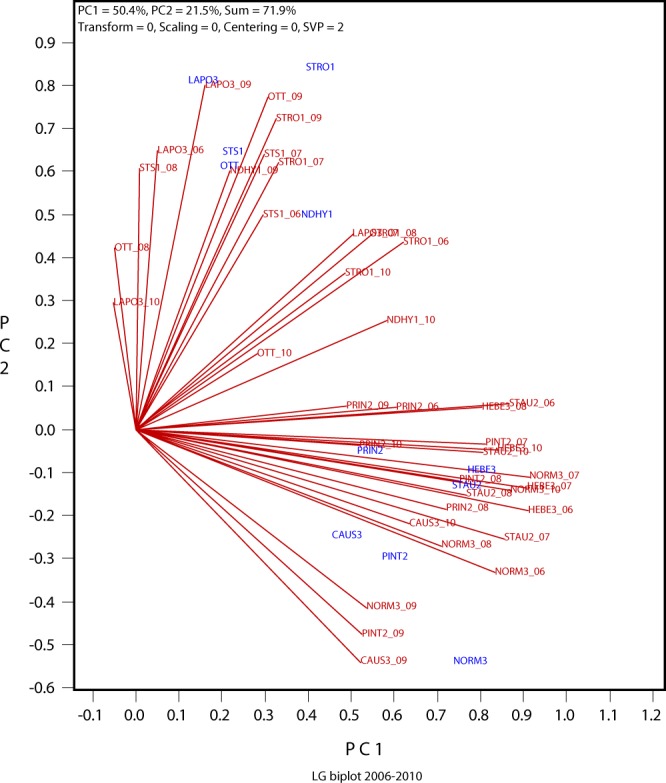
The LG (location-grouping) biplot displaying the correlations between locations in each of the five years (2006 to 2010) (Table 1). No scaling or centering was applied (“Scaling = 0”, “Centering = 0”) before subjecting the correlation table to singular value decomposition so that the biplot approximates the correlation values. The singular values were partitioned entirely to the location-year vectors (“SVP = 2”). The rows in Table 1 (trials) are presented in red and as location-year combinations. For example, OTT_10 means the trial at Ottawa in 2010. The columns in Table 1 (the locations) are presented in blue. See Table 1 for full location names.

The LG biplot (Fig. 2) is a graphical presentation of the yearly correlations among locations in Table 1. It may be viewed as a chart that stacks the location markers from each of the five GGE biplots (Fig. 1), with the location-year markers aligned according to their interrelations. The yearly patterns shown in Fig. 1 were largely retained in Fig. 2. An added function of the LG biplot is that it allows visualization of the similarity (repeatable GE) and variability (unrepeatable GE) of a location in correlation with other locations across years, a function similar to the GGE-GGL biplot^2^.

**Figure 3:**
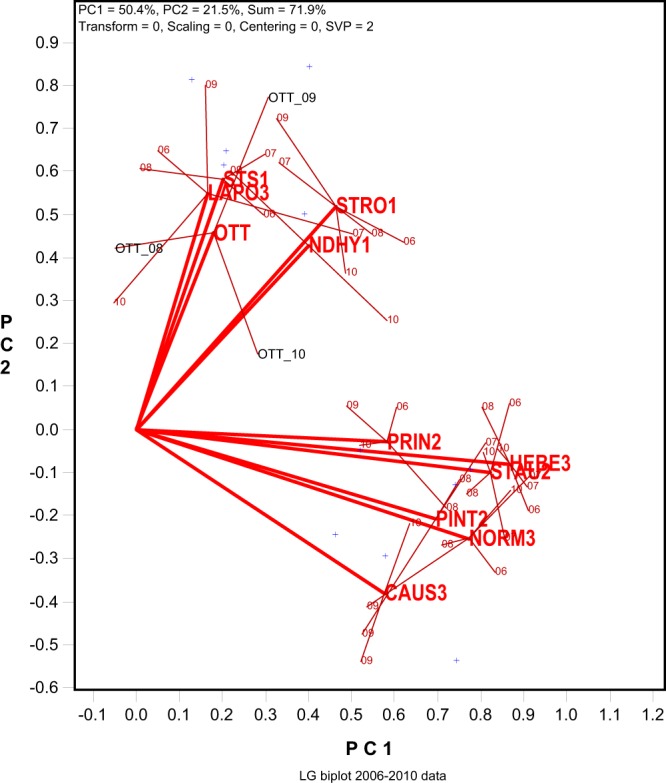
The mega-environment view of the LG biplot in Fig. 2. The placement of a location is determined by the mean coordinates of all trials conducted at the locations. For example, the placement of “OTT” was determined by the placements of the three trials conducted at Ottawa in 2008, 2009, and 2010, i.e., OTT_08, OTT_09, and OTT_10. See Table 1 for full location names. The makers for the columns in Table 1 are presented as “+” for clarity.

A more functional form of the LG biplot is presented in Fig. 3. It is the same biplot as in Fig. 2 but shows the mean placement of each location and its distribution in different years. The placement of a location was determined by the mean coordinates of all trials conducted at the location, as was done in the GGE-GGL biplot^2^. For example, the placement of “OTT” was determined by the placements of the three trials conducted at Ottawa in 2008, 2009, and 2010, indicated by OTT_08, OTT_09, and OTT_10, respectively (Fig. 3). The following information can be visualized from Fig. 3:

First, the 11 locations involved in the 2006 to 2010 trials fell into two distinct groups (mega-environments); the GE between the two mega-environments were, therefore, repeatable GE. The first group (the southern mega-environment) included five locations: three Quebec Zone-1 locations (NDHY1, STRO1, and STS1), a Quebec Zone-3 location (LAPO3), and the Ottawa Ontario location (OTT). The other group (the northern mega-environment) included six Zone-2 or Zone-3 locations (PINT2, PRIN2, STAU2, CAU3, HEB3, and NORM3). This is fully consistent with the conclusion based on GGE-GGL biplot analysis^2^. The grouping of LAPO3 with the Zone-1 locations demonstrates the superiority and essentiality of mega-environment delineation based on repeatable GE patterns.

Second, the correlations between trials (location-year combinations) from different mega-environments were mostly negative, ranged from highly negative (wide obtuse angles) to highly positive (small acute angles); as a result, the two mega-environments were slightly negatively correlated. This indicates that different cultivars must be selected and recommended specifically to each mega-environment. Alternatively, a “super” cultivar must be developed that is best for both mega-environments.

Third, large yearly variation existed in the placement of each location in the biplot (see the OTT location as an example). As a result, two locations in the same mega-environment may not be closely correlated every year even though they rank genotypes similarly across years. The sum of the yearly variations for individual locations within a mega-environment represents the unrepeatable GE. Its presence demands multi-year multi-location tests to identify superior and stable cultivars for the mega-environment. Its magnitude determines how many years and locations are needed for reliable genotype evaluation^3^.

**Figure 4:**
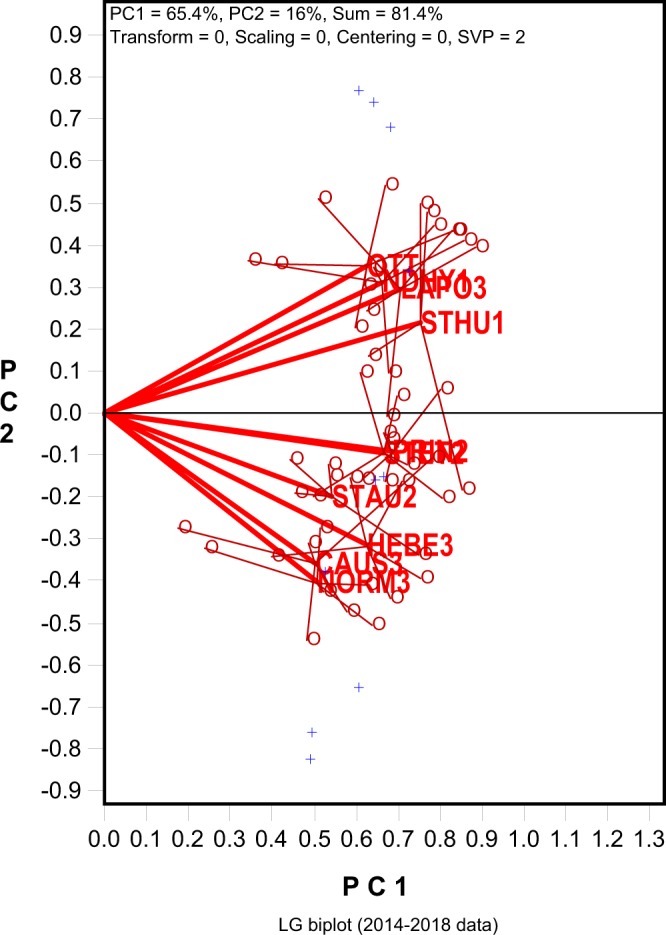
The LG (location-grouping) biplot to show two groups of locations based on the yield data from the 2014-2018 Quebec oat trials. This biplot was generated and can be interpreted the same way was the one in Fig. 3. The full names of the locations involved in the 2014 to 2018 trials are: Causapscal (Zone-3), HEBE3: Hebertville (Zone-3), LAPO3: (La Pocatière (Zone-3), NDHY1: N-D-de-St-Hyacinthe (Zone-1), NORM3: Normandin (Zone-3), OTT: Ottawa (Ontario), PRIN2: Princeville (Zone-2), STAU2: St-Augustin (Zone-2), STET2: St-Etienne (Zone-2), and STHU1: St-Hugues (Zone-1).

The LG biplot based on the grain yield data from the 2014 to 2018 Quebec oat trials (Fig. 4) supports the conclusions from the 2006 to 2010 data (Fig. 3). Despite dramatic breeding progresses and possible climate changes occurred between the two periods, the two location groups observed in Fig. 3 remained obvious in Fig. 4. The first group (the southern mega-environment) consisted of NDHY1, STHU1, LAPO3, and OTT, and the second group (the northern mega-environment) consisted of three Zone-3 locations (CAUS3, HEBE3, and NORM3) and three Zone-2 locations (STAU2, PRIN2, and STFR2). A difference is that the two mega-environments tended to be positively correlated in Fig. 4, as opposed to the negative correlation in Fig. 3. This was because the introduction of some more widely adopted cultivars such as Nicolas and Akina in recent years (see more in Discussion). Note that the Zone-3 location LAPO3 again fell with the southern locations (NDHY1, STHU1, and OTT), rather than with the other Zone-3 locations.

## Discussion

### Implication of mega-environment delineation

**Figure 5:**
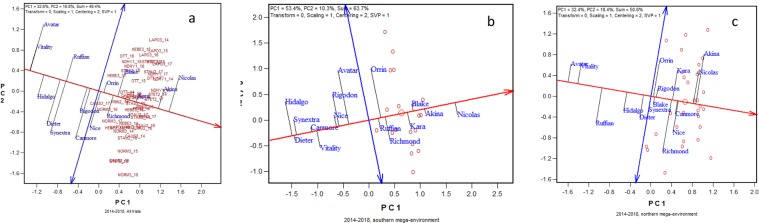
GGE biplots to show the mean yield and stability of 15 oat cultivars from 2014 to 2018 across (**a**) all locations, (**b**) southern mega-environment only (represented by NDHY1, OTT, LAPO3, and STHU), and (**c**) northern mega-environment only (represented by HEBE3, NORM3, CAUS3, STAU2, PRIN2, and STET2). The biplot was based on environment-standardized yield data (“Scaling = 1, Centering = 2”). See Fig. 4 for the full location names.

The purpose of mega-environment delineation is to utilize repeatable GE in plant breeding and crop production. This is achieved by breeding and deploying specifically adapted crop cultivars according to mega-environments. The GGE biplots presented in Fig. 5, which approximates the grain yield data of 15 oat cultivars tested throughout 2014 to 2018, illustrates this point. When viewed across all trials conducted during 2014 to 2018 (i.e., the whole region), the highest yielding cultivars was Nicolas, closely followed by Akina (Fig. 5a). The arrow on the single-arrowed line (the average environment axis or AEA) points to higher mean yield across trials, and the arrows on the double-arrowed line point to higher instability across trials^10^. Figure 5a shows that Nicolas yielded very well in trials above the AEA such as LAPO3_14 but not so well in trials below the AEA such as NORM3_18. When the yield data were summarized by mega-environment, Nicolas showed outstanding mean yield and stability across all trials in the southern mega-environment, clearly better than any other cultivars (Fig. 5b). Therefore, Nicolas can be recommended without hesitation to this mega-environment. In the northern mega-environment, while Akina and Nicolas were still the highest yielders on average, they were not the highest yielders in about half of the trials. Instead, Nice, Canmore, and/or Richmond were the highest yielders in these trials(Fig. 5c). Thus, Nice, Canmore, and Richmond showed contrasting responses to the environments within this mega-environment, in comparison with Akina and Nicolas (Fig. 5c). Therefore, to stabilize the oat yield in this subregion within and across years, Nicolas, Akina, Nice, Canmore, and Richmond should all be recommended to buffer the large and unpredictable GE. Note that Nice and Canmore yielded only about average across all trials (Fig. 5a) and yielded among the poorest in the southern mega-environment (Fig. 5b). Considering that the northern mega-environment is a key oat production area in Quebec, this understanding has important implications on oat production in Quebec.

### An alternative LG biplot

**Figure 6:**
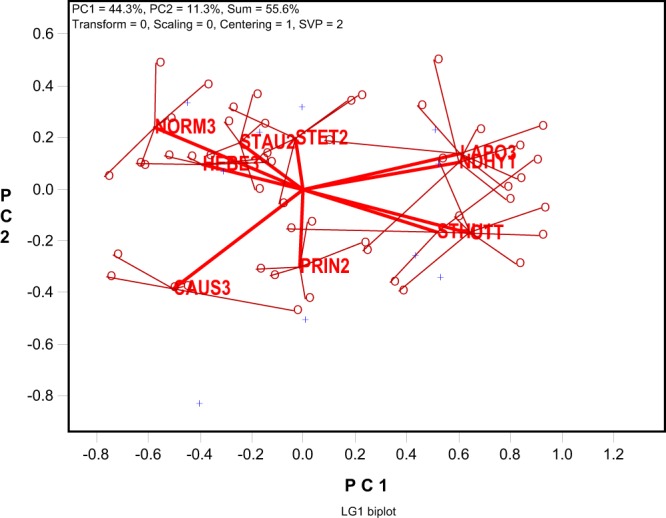
The LG1 biplot based on the same correlation table on which the LG biplot in Fig. 4 was generated. It differs from the LG biplot in Fig. 4 only in that the correlation table was centered by the grand-mean before subjecting to singular value decomposition, which is indicated by “Centering = 1”.

The model setting of no-scaling and no-centering (“Scaling = 0” and “Centering = 0”) in the LG biplots (Figs 2–4) allows visualization of the correlation coefficients between locations or trials (such as Table 1). When the LG biplot does not adequately display the correlation table, or when separating the locations, rather than visualizing the correlations between locations, is the focus, a LG biplot based on grand-mean centered correlation values (“Scaling = 0” and “Centering = 1”), referred as the “LG1 biplot”, may be used as a supplement. This LG1 biplot may be able to reveal the dissimilarities among locations that are masked by inclusion of the grand mean in the LG biplot based on un-centered correlation values. Presented in Fig. 6 is the LG1 biplot based on the 2014 to 2018 dataset. Instead of showing two groups of mega-environments in the LG biplot (Fig. 4), it shows two subgroups of locations within the northern mega-environment (on the left side of the biplot): CAUS3 and PRIN2 versus the other locations. It must be noted that the LG1 biplot cannot be interpreted as approximating the correlations between locations or trials in ranking genotypes and should only be used only as a supplement to the LG biplot.

### Some pitfalls in LG biplot analysis

In the correlation table used to generate a LG biplot (e.g. Table 1), the correlation of a trial with itself was assigned to “1”. While this is logical and standard, it may create an artifact to suggest that each location is a mega-environment by itself when only a few (e.g. three) locations are included in the analysis. In fact, when there are only two or three locations, the relations among them can be easily understood from examining the yearly GGE biplots (e.g. Fig. 1) or the correlation table (e.g. Table 1), and there is no need to resort to a LG biplot.

Another issue that needs attention is that in LG biplot analysis genotypes are treated as random samples of a genotype population. When this assumption is violated, i.e., when only a few genotypes are tested each year or when tested genotypes are dramatically different between years, location grouping may be blurred due to unusually large within-location variability.

## Conclusions

Crop variety trials are probably the most well-funded agricultural research. Regardless of economic developmental levels and financial situations, crop variety trials are conducted every year in every region for every main crop, and abundant data have been accumulated for all crops and regions. Such data can be utilized in selecting genotypes and recommending cultivars as short-term goals and in understanding the target region and the test locations as long-term goals, which in turn facilitates achieving the short-term goals. The long-term goals have been hindered due to lack of methods in utilizing the abundant but highly unbalanced multi-year data. The LG-biplot methodology solves this problem. It allows utilization of existing variety trial data to identify repeatable GE patterns, to delineate mega-environments, and to understand the scope of unrepeatable GE at a location and within a mega-environment. Such information is crucial for improving plant breeding efficiency, crop cultivar deployment, and crop productivity. Use of LG biplot analysis will save tremendous time and resources in planning and conducting new experiments.

## Methods

### The sample data

Grain yield data from the 2006 to 2010 Quebec oat trials were used in this study. This is the same dataset described and used in^2^ to conduct mega-environment analysis using a GGE-GGL biplot approach. Briefly, yield data were available for 26, 29, 34, 35, and 35 oat genotypes tested at 7, 7, 8, 8, and 9 locations across Quebec plus Ottawa Ontario in 2006 to 2010, respectively. The test locations were chosen to represent the three ecological zones of Quebec, ranging from Zone-1 in the south to zone 3 in the north. Fifteen genotypes were common in all five years; more genotypes were common in adjacent years. The locations used to represent each zone varied across years (Table 1). To present this multi-year, multi-location data in a single GGE biplot, a missing value imputation method had to be employed^10^. Based on the GGE-GGL biplot, two distinct oat mega-environments were identified. One mega-environment (the southern mega-environment) consisted of all Zone-1 locations plus Ottawa and La Pocatière, the latter being a Zone-3 location, geographically; the other mega-environment (the northern mega-environment) consisted of all Zone-2 and Zone-3 locations except La Pocatière. GGE-GGL biplot analysis was possible because of the presence of a relatively large number of common cultivars between years, which allowed missing values to be imputed with some confidence. The use of this dataset in the current study was to show that the same mega-environment delineation can be achieved using the LG biplot approach, which does not require the presence of common genotypes across years.

The yield data from the 2014 to 2018 Quebec oat trials consisted of 49 trials involving 10 locations and 116 genotypes; the number of genotypes tested each year was 50, 49, 45, 45, and 44, respectively. This dataset was used to validate the results from LG biplot analysis of the 2006 to 2010 dataset and to demonstrate the importance of mega-environment delineation on cultivar selection and recommendation.

### Data standardization

GGE biplot analysis was conducted yearly for the yield data from the 2006 to 2010 Quebec oat trials (Fig. 1); it is used here to relate yearly GGE biplot analysis to cross-year LG biplot analysis. The genotype-by-environment data of grain yield were standardized before subjecting to singular value decomposition (SVD). The standardization was conducted by:

1 $${P}_{ij}=\frac{{T}_{ij}-{\bar{T}}_{j}}{{s}_{j}},$$

where *P*_*ij*_ is the standardized yield of genotype *i* in environment *j*, *T*_*ij*_is the original yield of genotype *i* in environment *j* in the genotype-by-environment table, ${\bar{T}}_{j}$ is the mean yield across genotypes in environment *j*, and *s*_*ij*_ is the standard deviation in environment *j*. This data standardization is denoted as “Scaling = 1” and “Centering = 2” in the GGE biplots (Figs 1 and 5).

### GGE biplot analysis

A GGE biplot (Figs 1 and 5) approximates the genotypic main effect and GE of a genotype-by-environment two-way table^11^. It is based on the first two principal components (PC) resulting from subjecting the standardized genotype-by-environment table (*P*_*ij*_) to SVD. This process decomposes the table into genotype eigenvalues, environment eigenvalues, and singular values:

2 $${P}_{ij}=(d{\lambda }_{1}^{\alpha }{\zeta }_{i1})({\lambda }_{1}^{1-\alpha }{\tau }_{1j}/d)+(d{\lambda }_{2}^{\alpha }{\zeta }_{i2})({\lambda }_{2}^{1-\alpha }{\tau }_{2j}/d)+{\varepsilon }_{ij},$$

where *ζ*_*i1*_ and *ζ*_*i2*_ are the eigenvalues for PC1 and PC2, respectively, for genotype *i*; *τ*_*1j*_ and *τ*_*2j*_ are the eigenvalues for PC1 and PC2, respectively, for environment *j*, and ${\varepsilon }_{ij}$ is the residual from fitting PC1 and PC2 for genotype *i* in environment *j*; λ_1_ and λ_2_ are the singular values for PC1 and PC2, respectively. α is the singular value partitioning (SVP) factor. When α = 1 (i.e., SVP = 1), the biplot is said to be genotype-focused, and is suitable for comparing genotypes (Fig. 5). When α = 0 (i.e., SVP = 2), the biplot is said to be environment-focused, and is suitable for visualizing correlations among environments (Fig. 1). The scalar *d* is chosen such that the length of the longest vector among genotypes equals to that among environments; this is important for generating a functional biplot^10^. The GGE biplot was constructed by plotting $({d{\rm{\lambda }}}_{{\rm{1}}}^{{\rm{\alpha }}}{{\rm{\tau }}}_{{\rm{i1}}})$ against $({d{\rm{\lambda }}}_{{\rm{2}}}^{{\rm{\alpha }}}{{\rm{\tau }}}_{{\rm{i2}}})$ for genotypes and plotting $({{\rm{\lambda }}}_{1}^{{\rm{1}}-{\rm{\alpha }}}{\tau }_{{\rm{ij}}}/{\rm{d}})$ against $({{\rm{\lambda }}}_{2}^{{\rm{1}}-{\rm{\alpha }}}{\tau }_{{\rm{2j}}}/d)$ for environments in the same plot.

### LG biplot analysis

LG biplot analysis includes two steps. First, the yearly Pearson correlations among test locations across tested genotypes were calculated to form a location by trial table of correlations like Table 1. Second, this table was subjected to SVD and displayed in a LG biplot. The process of generating a LG biplot is the same as generating a GGE biplot (equation 2) except to define *P*_*ij*_ as the Pearson correlation coefficient between location *i* and location-year combination *j* (Table 1) and replace “genotypes” with “locations” and “environments” with “trials” or “location-year combinations”.

The LG biplot was so named because both the rows and the columns of the correlation table (Table 1), and their makers in the biplot (Figs 2–4), are locations, and the purpose was to visualize the similarity/dissimilarity among locations in ranking genotypes. No scaling or centering was performed before subjecting the correlation table to SVD (denoted as “Scaling = 0” and “Centering = 0”). The LG biplot, therefore, approximates the correlation values in Table 1. Prior to SVD, any missing values in Table 1 were estimated using a method described in^11^. Note that no information is required about the tested genotypes in LG biplot analysis; they are treated as random samples in the population of genotypes.

## Data Availability

All necessary data are included in the manuscript.
